# Bridging Cultures: A Cambodian Psychiatrist’s Perspective on Psychiatry and Addiction Exchange Programs in the Netherlands and South Africa

**DOI:** 10.1192/j.eurpsy.2025.2379

**Published:** 2025-08-26

**Authors:** R. Ngin, S. Bora

**Affiliations:** 1 Mental Health, Cambodian’s Children Fund, Phnom Penh, Cambodia

## Abstract

**Introduction:**

This knowledge exchange program was designed to enhance the clinical training of a Cambodian psychiatrist in general psychiatry and addiction care. Conducted between July 1, 2024, and August 30, 2024, the program involved immersive clinical experiences in the Netherlands and South Africa. The exchange facilitated engagement with leading mental health institutions, offering valuable insights into advanced psychiatric practices and addiction care models.

**Objectives:**

The primary objectives of the program were to improve clinical competencies in diagnosing and managing psychiatric and addiction disorders, to gain exposure to multidisciplinary and community-based care models, and to develop cultural competence in adapting psychiatric practices to low-resource settings like Cambodia. These goals were pursued through a combination of clinical visits and participatory learning activities.

**Methods:**

The psychiatrist participated in clinical visits and hands-on learning at Radboud University Medical Center and Pro Persona in the Netherlands, as well as Rustenburg Addiction Care in South Africa. These experiences included observing advanced treatments and participating in community-based psychiatric care programs. The program’s structure combined hands-on training and multidisciplinary teamwork to provide a comprehensive learning experience.

**Results:**

The program yielded significant improvements in the psychiatrist’s ability to manage complex addiction cases and implement community-based care. Additionally, the experience fostered greater cultural competence, enabling the application of psychiatric practices suited to the Cambodian context. The collaboration also promoted potential future psychiatric training initiatives and international partnerships.

**Conclusions:**

The knowledge exchange program was successful in enhancing the psychiatrist’s clinical skills and cultural awareness. It underscored the importance of international collaboration in psychiatry and addiction care, providing a model for how mental health professionals from low-income countries can benefit from exposure to global best practices and community-based care strategies.

**Disclosure of Interest:**

None Declared

